# Laser Ablated Carbon Nanodots for Light Emission

**DOI:** 10.1186/s11671-016-1638-8

**Published:** 2016-09-22

**Authors:** Delfino Reyes, Marco Camacho, Miguel Camacho, Miguel Mayorga, Duncan Weathers, Greg Salamo, Zhiming Wang, Arup Neogi

**Affiliations:** 1 Department of Physics, University of North Texas, Denton, TX USA; 2 Facultad de Ciencias, Universidad Autónoma del Estado de México, Instituto Literario 100, Col. Centro, C.P. 50000 Toluca, Estado de México México; 3 Laboratorio de Investigación y Desarrollo de Materiales Avanzados, Facultad de Química, Universidad Autónoma del Estado de México, Campus Rosedal, Km 14.5 Carretera, Toluca-Atlacomulco, San Cayetano de Morelos C.P. 50925 México; 4 Laboratorio de Fotomedicina, Biofotónica y Espectroscopía Láser de Pulsos Ultracortos, Facultad de Medicina, Universidad Autónoma del Estado de México, Jesús Carranza y Paseo Tollocan s/n, Toluca, C.P. 50120 México; 5 Department of Physics, University of Arkansas, Fayeteville, AR USA; 6 Institute of Frontier and Fundamental Sciences, Univ. of Engineering Science and Technology, Chengdu, China

**Keywords:** Laser ablation, Carbon nanodots, Photoluminescence

## Abstract

The synthesis of fluorescent carbon dots-like nanostructures (CNDs) obtained through the laser ablation of a carbon solid target in liquid environment is reported. The ablation process was induced in acetone with laser pulses of 1064, 532, and 355 nm under different irradiation times. Close-spherical amorphous CNDs with sizes between 5 and 20 nm, whose abundance strongly depends on the ablation parameters were investigated using electron microscopy and was confirmed using absorption and emission spectroscopies. The π- π* electronic transition at 3.76 eV dominates the absorption for all the CNDs species synthesized under different irradiation conditions. The light emission is most efficient due to excitation at 3.54 eV with the photoluminescence intensity centered at 3.23 eV. The light emission from the CNDs is most efficient due to ablation at 355 nm. The emission wavelength of the CNDs can be tuned from the near-UV to the green wavelength region by controlling the ablation time and modifying the ablation and excitation laser wavelength.

## Background

Carbon dots- or wire-like nanostructures comprise of carbon structures exhibit quantum confinement when one dimension is less than 10 nm while carbon nanodots (CNDs) ascribed to almost spherical nanoparticle is a topic of extensive research interest. Carbon-based light emitting nanoparticles such as carbon nanotubes [1], graphene or graphene oxide [2, 3], graphene quantum dots [4, 5], carbon quantum dots [6], carbon nanodots-like nanoparticles [5, 7] have been reported to be ideal due to their stability for various biomedical applications. Carbon-based emitters are chemically inert resulting in low toxicity compared to other inorganic colloidal nanoscale light emitters [2]. The ease of functionalization to carbon makes it biocompatible, and carbon-based nanostructures are increasingly being used for various biomedical applications such as bioimaging, disease detection, sensing, or drug delivery [5–8]. Various techniques are currently being used to synthesize these carbon-based emitters which affect their stability, toxicity, and emission efficiency due to modification in their crystalline and surface properties [5].

CNDs can be synthesized in the form of nanoparticles without a perfect crystal lattice (amorphous) or high crystalline quality structures such as well-defined quantum dots (CQDs) [5]. The main difference between CQDs and CNDs is that the size can be tuned to modify the emission properties due to quantum confinement in CQDs [9] whereas CNDs need modification of surface properties by surface passivation techniques to stabilize the optical properties and allows one to tune its PL features [5, 10]. Due to crystalline defects and surface recombination, the emission bandwidth is much wider for CNDs than for CQDs [5, 9].

CNDs were firstly reported as a by-product of single-walled carbon nanotubes (SW-CNTs) fabricated by arc-discharge method. SW-CNT complex compound could be separated into various one lower dimensional carbon nanostructures with size-dependent fluorescent properties [11]. Following the initial report, the origin of light emission from these structures has been extensively studied [5, 7, 9, 12]. CNDs have been synthesized by various techniques. These include chemical techniques such as combustion or hydrothermal routes [5, 13], electrochemical synthesis [6, 9], catalytic synthesis methods [14], and electric or electromagnetic techniques such as microwave-aided synthesis [15], arc discharge [11, 12], and laser ablation. The target for the laser ablation included either solid targets [16–19] in liquid (LASL) or vapor [20]. There have been reports of using larger carbon microparticles as flakes, powders, and multiwall-CNT as targets [21–24]. Chemical techniques are usually time-consuming and need careful control of the chemical pathway for high-quality emitters. LASL on the other hand is an efficient and quick process to synthesize high-quality CNDs with [16, 17] or without surface passivation [18].

The potential of synthesizing CNDs through laser ablation in solution is limited by a lack of information regarding the correlation of the physical parameters that affects the size and optical properties. There are recent reports of the influence of laser wavelength and power on the synthesis of metal nanoparticles [25], but not a comprehensive study in CNDs. In particular, the LASL technique (synthesis of CND using laser ablation of solid targets in liquids) is fairly versatile due to the different parameters that can be modified and is not limited to the features of the target or liquid media, fluence, wavelength, repetition rate, or pulse duration of the incident laser. The variation of laser wavelengths and excitation parameters of the laser [16–19] for the optimization of the LASL has been shown to be used as fiber optical sensor [16]. The size distributions can vary between 1.5 and 3 nm [22] in ethanol or similar ionic liquids, when the solid target is ablated to produce carbon microparticles and can also result in fragments of MWCNTs, fluorescent CNDs, or carbon nanostructures [21–24]. The LASL technique for the production of fluorescent CNDs can be very fast and efficient as a large volume can be produced is a short-time duration [16–19]. LASL offers a significant advantage over other synthesis techniques such as the ablation of suspended microparticles or powders [21, 22], which requires longer ablation time for synthesis of high quality or smaller-sized nanostructures [23]. A careful control of heat dissipation via wavelength and intensity variation can also result in a greater control of the size distribution in this technique. In this present work, the size of the emitting nanoparticles synthesized is in the nanoscale range compared to microscale-ablated particles in the previous reports [21, 22].

Motivated by the need to realize efficient light-emitting carbon nanodots, this work reports the optimum conditions to synthesize CNDs obtained by laser ablation in solution technique. The light emission efficiency of CNDs has been observed to depend on the excitation wavelength and the irradiation time of laser irradiation used for the ablation process. Morphological features of the CNDs were studied using TEM which showed that the ablation rate depends on the physical parameters that make it possible to synthesize CNDs that are nearly spherical in shape and has dot diameter less than 5 nm. Absorption and emission spectroscopies were performed to confirm the synthesis of light-emitting carbon nanostructures.

## Methods

### Carbon Dots-Like Nanoparticles (CDNs) Solution Synthesis

**Fig. 1:**
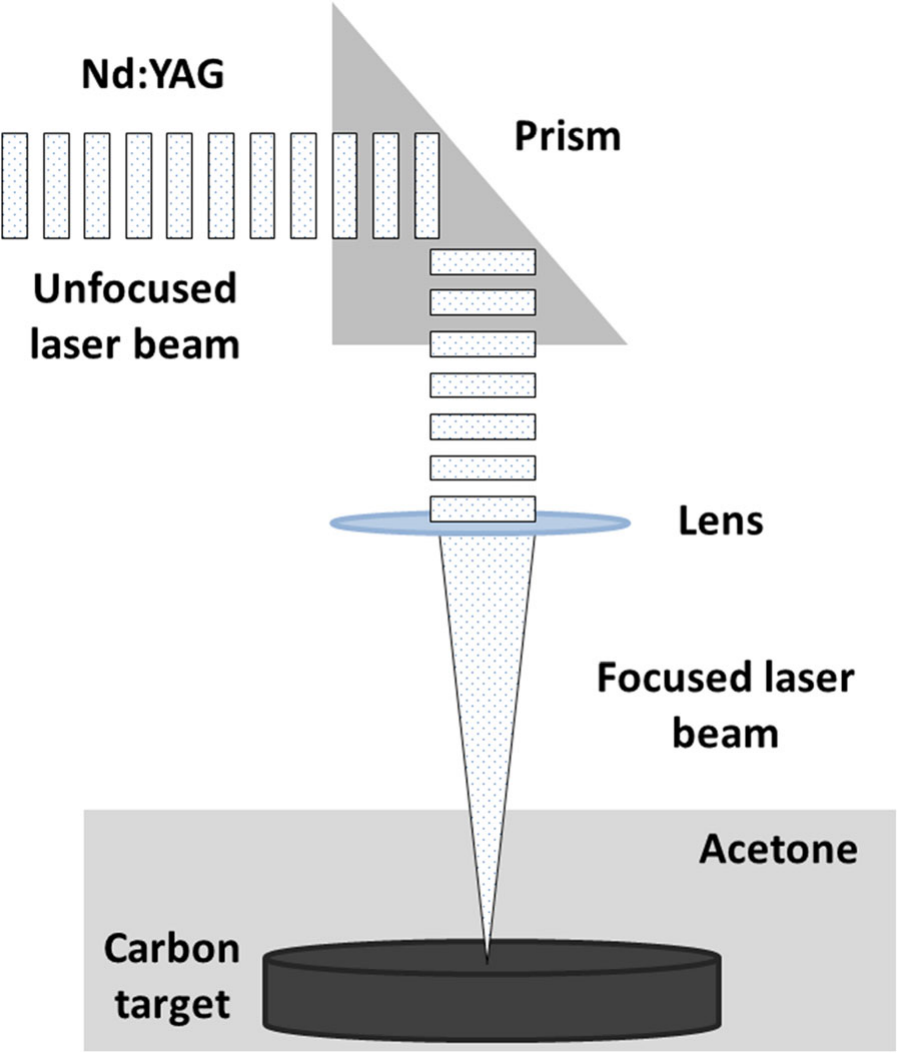
Experimental design. The experimental setup used for the synthesis of the CDNs samples. Nd:YAG laser with harmonic generation allows the ablation of the carbon target at 1064, 532, and 355 nm

CNDs were synthesized by ablating a solid carbon target (Kurt J. Lesker Co., 99.999 % pure, 2.54 cm × 0.375 cm) immersed in a beaker with 12 ml of pure acetone (Sigma-Aldrich Co.) using nanosecond (ns) laser pulses. Ns pulses from a Nd:YAG laser (Quanta Ray-NCR) were focused on the target surface (Fig. 1). The unfocused spot laser diameter at the output of the laser was 0.64 cm with a repetition rate of 10 Hz. The beaker was sonicated to avoid laser ablation at the same place and to avoid any changes in the input flux density due to the change in the focusing of the laser spot size.

The effect of the laser wavelengths on the ablation process and its influence on the optical properties of the carbon nanodots were performed using three different wavelengths, i.e., the fundamental (1064 nm), the second (532 nm), and third (355 nm) harmonic emission of the Nd:YAG laser. Due to the nonlinear harmonic generation process, the pulse widths of the incident laser pump vary from 7 ± 2 to 5 ± 2 ns as the 1064 nm fundamental is tripled to 355 nm. The CNDs solutions were synthesized using these three laser wavelengths with various irradiation times (150, 300, 600, and 900 s). The fluence for the three wavelengths was maintained at a constant value of 12.5 ± 0.5 J/cm^2^ by varying the spot area (on the carbon target surface) after the lens used for focus.

For each wavelength, the focusing was induced using different lenses, designed specifically for a particular wavelength. Samples using the 1064-nm laser light were produced using an infrared lens that had a spot diameter of 0.10 cm. The samples produced at 532 or 355 nm wavelength used visible and UV lens with a final spot diameter of 0.10 and 0.06 cm, respectively. The selected spot areas were ablated at the same laser fluence. The laser ablation experiments were performed at environment conditions without any control, 20 °C and 1 atm. The size and shape were characterized using 35-kV transmission electronic microscopy (TEM). The sample was prepared by evaporating a drop of the CND solution on a copper grid.

### Optical Characterization

Absorption spectroscopy was performed using an Agilent spectrometer (Agilent Technologies, 8453) with the CND solution in a 1 × 1 cm quartz cuvette with two transparent facets. All spectra, including the reference from pure acetone solution were measurement from 300 to 900 nm. Photoluminescence spectrum was measured using a spectrofluorophotometer (Shimadzu, RF-5301), with the sample in quartz cuvettes having four transparent facets. Samples were excited at various different wavelengths (369, 390, 410, 430, and 450 nm). Time-resolved photoluminescence spectroscopy was performed by exciting the samples with Ti:Sapphire laser with a 80-fs pulsed laser and a repetition rate at 80 MHz. The fundamental laser source was doubled to obtain a laser wavelength at 345 nm for the excitation of the samples. The time-resolved PL intensity was dispersed through a spectrometer, and the spectrally resolved PL signal was collected using a Hamamatsu streak camera.

## Results and Discussion

**Fig. 2:**
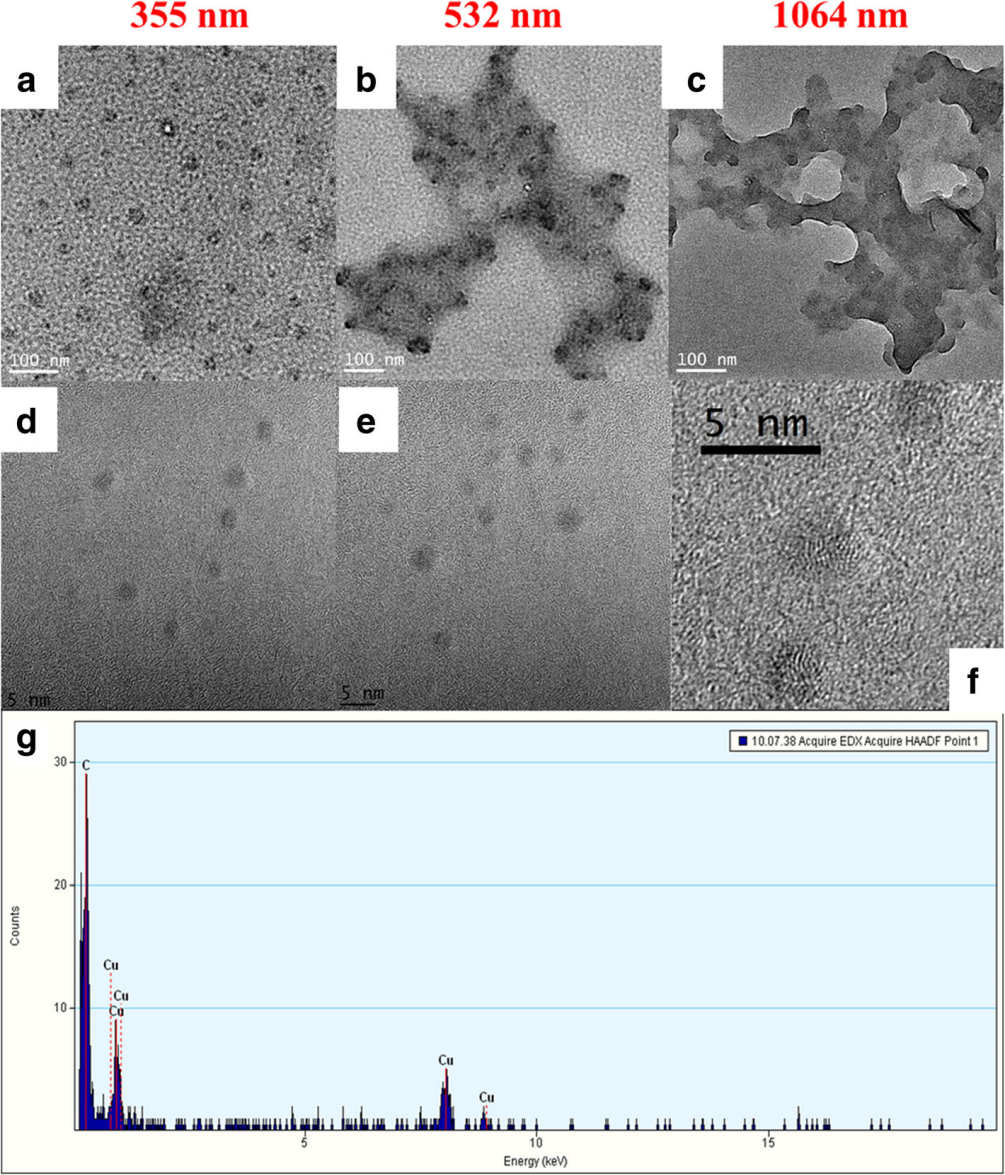
TEM pictures of the CNDs. **a** 355 nm. **b** 532 nm. **c** 1064 nm. **d–f** HRTEM pictures, CNDs, or sizes less than 5 nm are observed. **g** Displays the EDS, showing the abundance of carbon

Figure 2 shows the TEM images of the CNDs obtained with 355 nm (a), 532 nm (b), and 1064 nm (c). In these images, it can be observed that for ablation at 532 nm and 1064 nm, there are a large amount of CNDs which were agglomerated and large clusters were observed on the TEM grid. In the samples that were ablated using the 355 nm (a), smaller size of the agglomerates were observed. These means that in 355 nm, the amount of carbon material removed from the target is lesser than the other two, which reduce the size of the agglomerates. As will be seen below, this difference significantly impacts the optical properties and the light emission from the CNDs solutions. A high-resolution TEM (HRTEM) analysis of the carbon samples allows the identification of well-defined spherical carbon nanostructures with sizes below 3 nm, which can be classified as carbon quantum dots (Fig. 2d–f). The image in Fig. 2f shows the crystalline lattice structures which corresponds to carbon atoms, which is also confirmed by the EDS spectrum shown in Fig. 2g. CNDs with 5-nm sizes were produced via laser ablation of a carbon target in the presence of water vapor with argon as the carrier gas required more detailed experimental investigation compared with the simple route reported here [12].

**Fig. 3:**
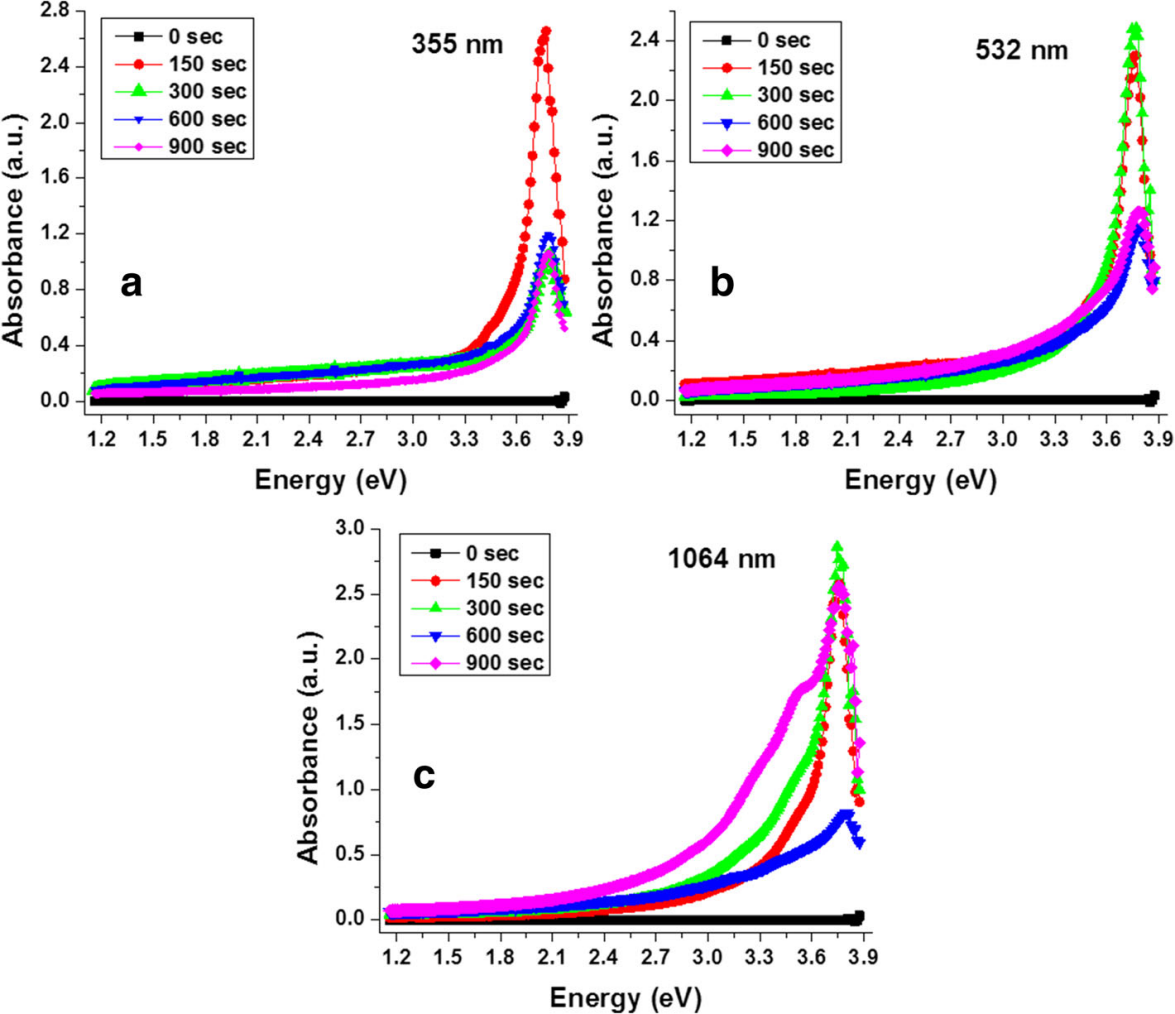
Absorbance features of the CNDs-based solutions. Absorbance spectrums of the CNDs solutions for the three wavelengths used for its synthesis (**a** 355, **b** 532, and **c** 1064 nm)

Absorbance spectra of the CNDs solutions for the different irradiation times (150, 300, 600, and 900 s) at various wavelengths used for its synthesis are displayed in Fig. 3 (a 355, b 532, and c 1064 nm). These plots depict the measured optical absorbance from 1.15 eV (1070 nm) to 3.87 eV (320 nm). The spectrum of the reference solution (pure acetone solvent) is drawn as a black scatter line for comparison and shows no absorption states in the region of interest. It is well known that pure acetone has at least two high-energy absorption peaks at 6.63 (187 nm) and 4.60 eV (270 nm) due to the π→π* and *n*→π* electronic transitions [26], respectively. From Fig. 3, it can be seen that there is no appreciable absorptive states for photon energies lesser than 3.0 eV from any of the samples. However, large absorbance can be observed in the ultraviolet range, where all samples exhibit a non-Gaussian absorption peak identified around 3.76 eV (327 nm). This emission originates from the CNDs and is due to shifted π- π* electronic transitions of carbonyl groups, and has been showed that after a surface functionalization, this electronic transition is red-shifted 38–50 nm, which is in agree with the shifted position reported here [27]. Even when any post functionalization was done, during the laser interaction with the solvent, it could induce the attachment of dissociated acetone molecules on the CNDs, inducing a kind of surface functionalization of ketones, for example.

The 3.76-eV absorption peak was observed to be more intense and narrower for the shorter irradiation time (~150 s) of the solutions prepared with 355-nm laser irradiation (Fig. 3a). The ablation is confined to the surface layers due to the lower penetration depth of the laser into the dielectric. This results in a higher size uniformity which leads to narrower FWHM of the absorption spectrum. The narrower dot distribution at 355 nm and the small size of the dots synthesized are correlated to the TEM measurements. However, for CNDs synthesized with 532 (Fig. 3b) and 1064 (Fig. 3c) nm exhibit the maximum absorption when ablated for 150 and 300 s. It is also observed that for 355 and 532 nm laser irradiance, the absorbance considerably decreases with longer irradiation times. However, for 1064 nm, all spectrums are wider than for the other wavelengths as at infrared wavelengths, and the penetration of laser into the dielectric is deeper (>500 nm) that results in a wider size distribution of the synthesized nanoparticles during the ablation process. At a longer irradiation time, (obtained after 900 s), a well-defined shoulder appears at 3.54 eV (pink scatter line) which is relatively less prominent for the sample under 600-s irradiation. The shoulder located at 3.54 eV has been reported to be originated by the *n*-π* transitions [28]. The relatively broader spectral width is due to the agglomeration of CNDs observed in the solutions synthesized with 532 and 1064 nm as observed in TEM images in Fig. 2. The fact that the intensity of the 3.76 eV absorption peak shows a decrease or difference with respect to the shorter irradiation time, can be related with a change in the amount to the species responsible of the π- π* transitions due to the interaction between the laser beam and the CNDs already formed and modifies the surface features of the last ones.

**Fig. 4:**
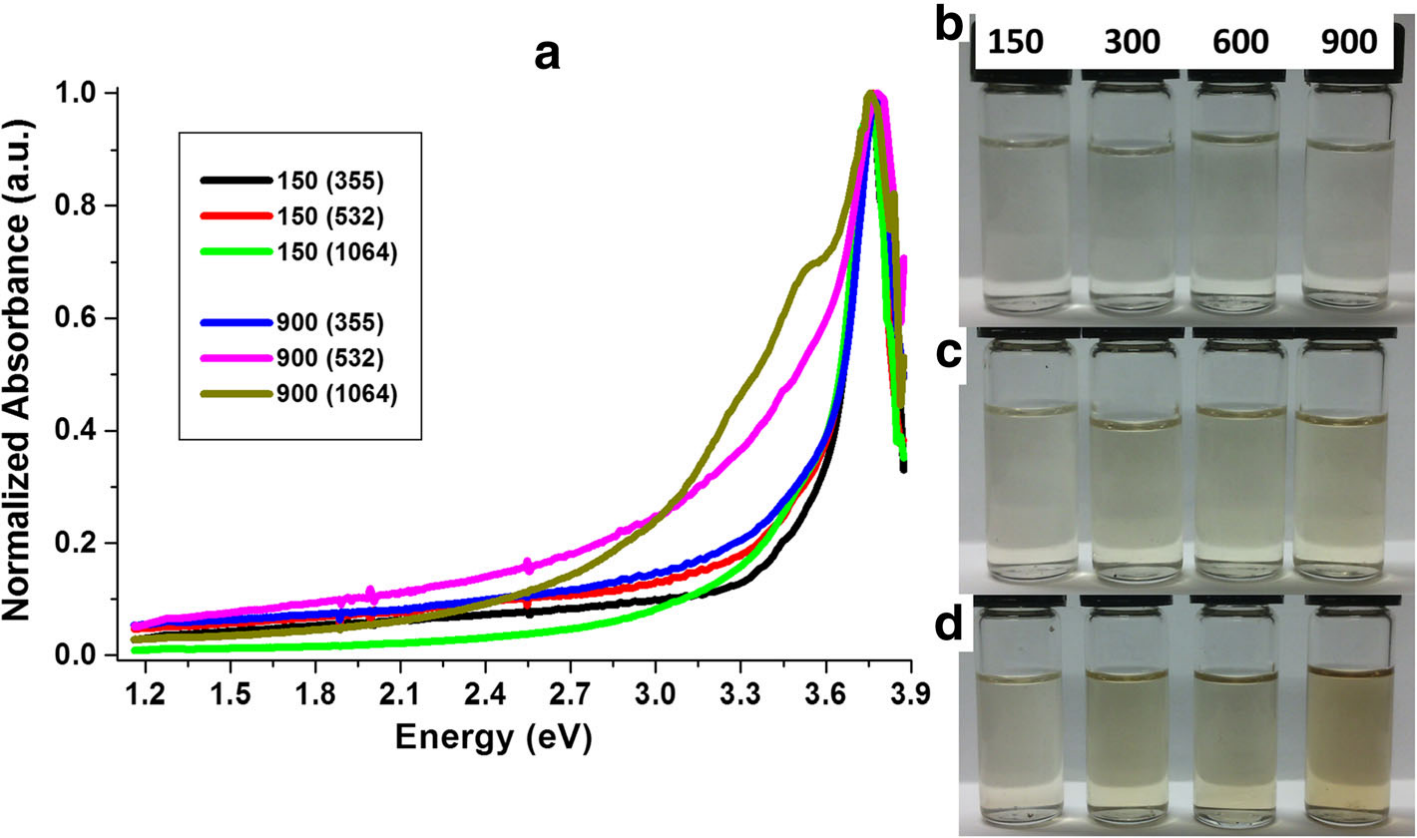
Analysis of the absorbance results. **a** Normalized absorbance of the solutions for 150 and 900 s of irradiation. Optical pictures of the CNDs solutions for the different irradiation times synthesized with **b** 355, **c** 532, and **d** 1064 nm

From the absorbance spectrums, it is evident that by increasing the irradiation time, the absorbance decrease, at least for samples prepared with 355 and 532 nm lasers, which seems to be contradictory. In order to clarify this observation, the normalized absorbance spectrums are displayed in Fig. 4a. It can be observed that in all cases, the width of the 3.76 eV peak is higher for the longer irradiation time, particularly for those samples obtained with 532 and 1064 nm. It is also evident that the absorption at lower energy increases with the irradiation time and can be correlated with the presence of larger CNDs due to presence of larger sized agglomerates as shown in Fig. 2. Optical images of the solutions under the different irradiation times for the 355, 532, and 1064 nm laser wavelength used in the synthesis are showed as inset labeled as Fig. 4b–d, respectively. From these optical images, it can be observed that the CNDs solutions tend to be more turbid at larger irradiation time. The increase in turbidity is more visible for those solutions synthesized with 532 and 1064 nm. The agglomeration can also result in enhanced scattering for laser ablation from deeper within the substrate and coalescence under thermal effect due to the absorption of light for longer irradiation time.

The major process during the laser-target interaction is the electron heating by the laser field. In laser ablation of solids in vacuum or gaseous medium, most of the laser energy is directly transferred to the solid substrate [29]. However, when it is performed in liquid environment, part of the energy carried by the laser beam is absorbed by the liquid before the transfer of energy to the solid surface which is ablated. The laser energy is also absorbed by the ablated material suspended in the liquid [25] during the ablation of the solid in case of longer wavelength excitation and longer ablation times. In our case, from Fig. 4a, it is possible to see that CNDs solutions are non-highly absorbent in the 1.14 to 2.4 eV, which correspond to the 1064 nm (1.16 eV) and 532 nm (2.33 eV) laser excitation. However, all the sample solutions are more absorbents between 3.45 and 3.8 eV, which include the 355 nm (3.49 eV) laser. This behavior impacts on the laser ablation efficiency for 355 nm but not for the other wavelengths, and this fact perfectly agrees with the previous TEM results.

The overlap of already formed NPs with incoming laser pulses occurs in the most ordinary laser ablation of solids in liquid experiments. It is well known that laser wavelength of the incident laser beam determines the skin depth, and as a consequence, ablation depth can be altered. It was reported for ablation of platinum in water that absorption of UV photons by the surface electrons via inter-band transitions is more uniform and leads to nice corrugation on the surface, whereas near-infrared radiation is preferentially absorbed by the defects and impurities of the material, consequently resulting in the formation of random surface structures [25, 29]. For the ablation process of carbon in vacuum, the ablation depth by a similar and highly focused laser beams (~300-μm diameter) was showed to be smaller for larger wavelengths due to the absorption coefficient of carbon decreases for longer wavelength [30, 31]. In the case of laser ablation in liquids, this behavior is driven by the liquid surrounding the target [25]. Essentially, the ablation depth (optical penetration or skin depth) *δ*, is given by the expression *δ* = √(2(*μ*_0_*σω*)^−1^, where *μ*_0_ is the permittivity, *ω* is the frequency of the impinging laser, and *σ* is the electrical conductivity. A simple calculation allows one to obtain the skin depth for the different wavelengths as, *δ*_1064_ ≈ 623 nm, *δ*_532_ ≈ 440 nm and δ_355_ ≈ 359 nm. It is necessary to point out that these results are different for ablation in liquids during the laser ablation process due to its interaction with the. The solvent absorption or scattering of light by the carbon nanostructures that are being formed during the ablation process additionally decrease the skin depth or penetration depth of the laser in the target dielectric. Considering that our samples are highly absorbent in the ultraviolet region, for the case of the samples obtained with 355 nm (3.49 eV), the skin depth is expected to be less than the estimate. The efficiency of the CNDs production by various lasers will be further implied by photoluminescence from the nanostructures.

**Fig. 5:**
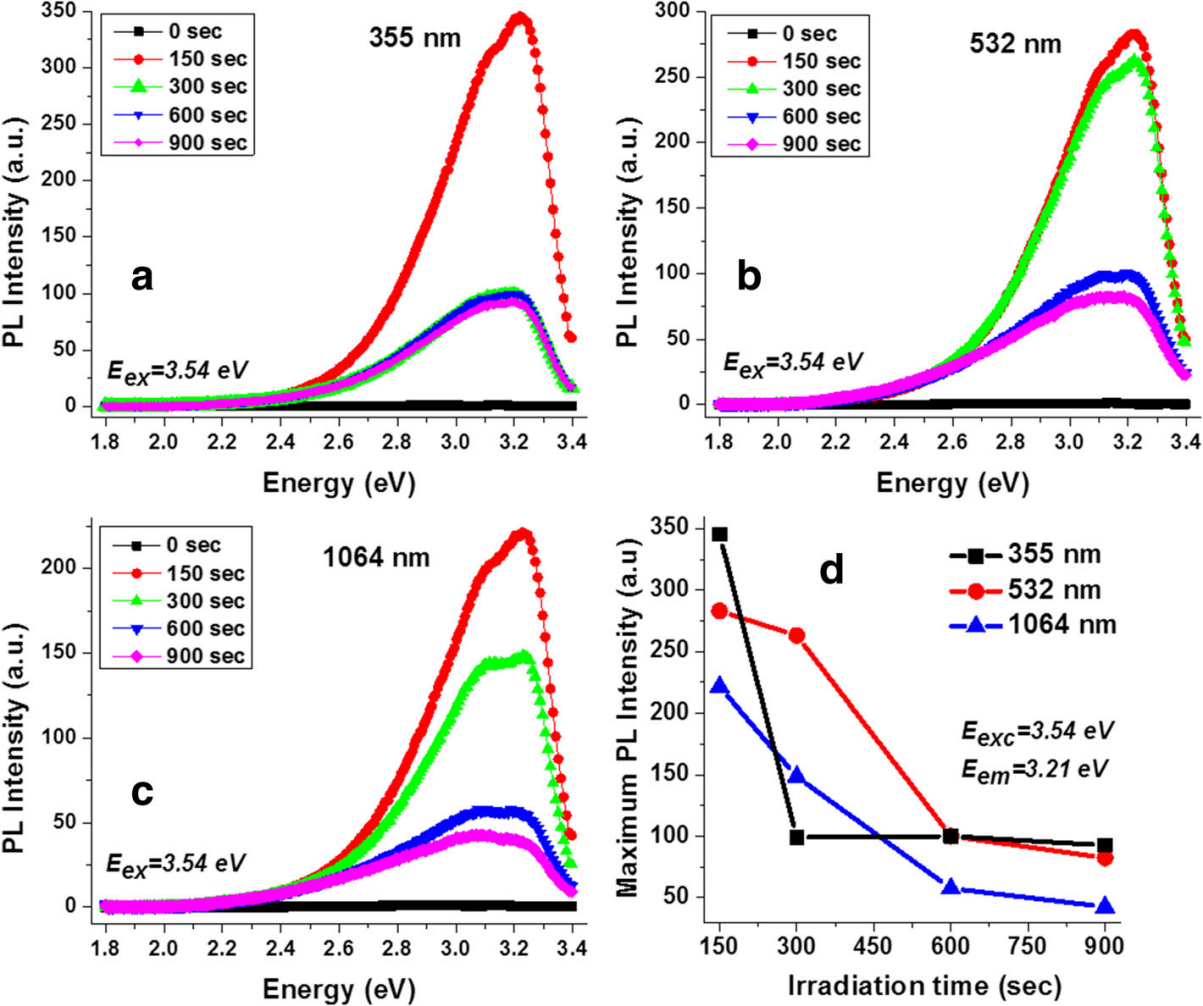
PL responses of the CNDs solutions. PL spectrums of the CNDs solutions synthesized with **a** 355, **b** 532, and **c** 1064 nm, exciting with 3.54 eV. **d** Maximum intensity recorded when exciting with 3.54 eV for each solution

The photoluminescence (PL) response of the CNDs solutions dependent on the irradiation time was analyzed by exciting all the samples with UV light of 3.54 eV (350 nm). Results are displayed out in Fig. 5 (a 355, b 532, and c 1064 nm). The PL emission of the reference pure acetone solution is also included (black scatters), and does not show any PL response. It can be seen that in all cases, the emission is characterized for a broad band ranging from 3.38 eV (365 nm) to 2.52 eV (490 nm) for the smaller irradiation time while, for the larger irradiation time, the full-width at half-maxima is widened and extends to 2.24 eV (550 nm). These results are in agreement with the previous reports in CNDs produced by laser fragmentation [21–23]. It can be observed from Fig. 5 that the intensity of the PL emission is decreased by increasing the irradiation time. Especially for the samples synthesized with 532 (Fig. 5b) and 1064 (Fig. 5c) nm, the PL intensity gradually decrease. For those obtained with 355 nm (Fig. 5c), there is a considerable decrease after 150 s and does not change significantly for the larger irradiation times, as can be seen in Fig. 5d, where the maximum intensity recorded at 3.21 eV for each irradiation times and wavelength of synthesis is plotted when exciting with 3.54 eV. The emission properties at 355 nm correspond to similar behavior for the absorption spectra depicted in Figs. 3 and 4.

**Fig. 6:**
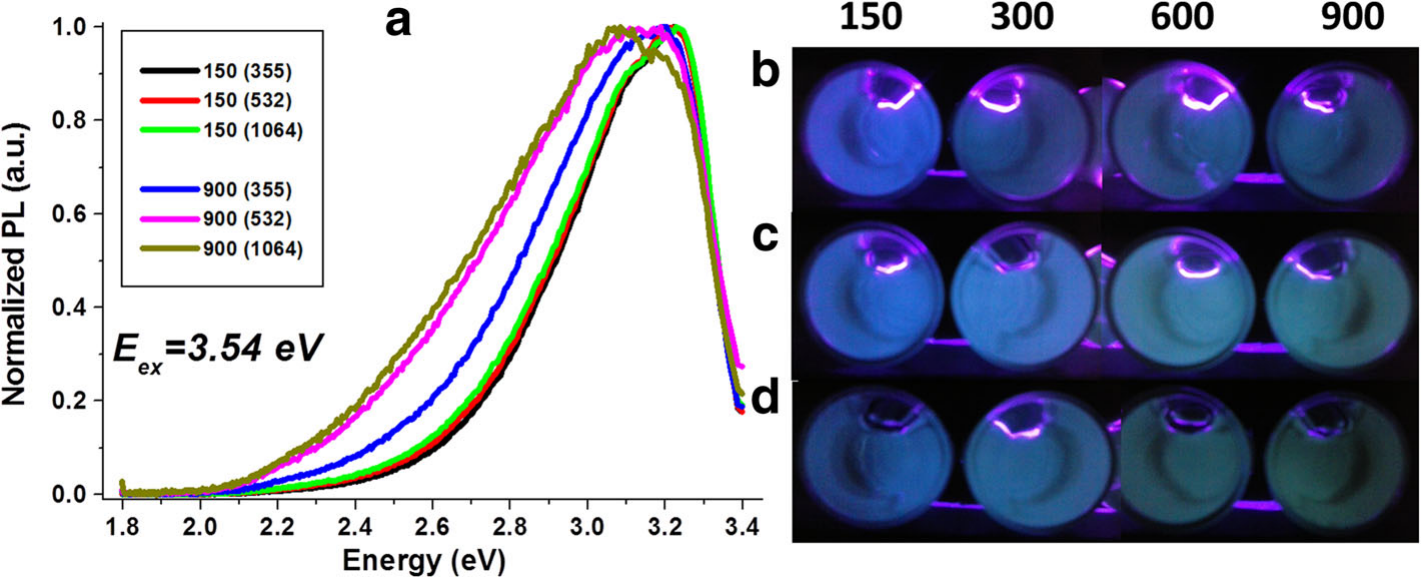
Analysis of the PL response. **a** Normalized PL spectrums of the solutions for 150 and 900 s of irradiation, exciting with 3.54 eV. CNDs solutions illuminated with an UV lamp, **b** 355, **c** 532, and **d** 1064 nm

A normalized PL spectrum shown in Fig. 6 demonstrated the effect of the irradiation time on emission properties of the CNDs and on the optimized condition required for the synthesis process. For a shorter irradiation time (150 s), with a 3.54 eV excitation source, (Fig. 6a), the PL spectra are very similar, and the PL emission width is independent of the wavelength used for the synthesis process. However, the FWHM of the PL increases with the ablation time (~0.2 eV), and the emission peak is red-shifted with the 1064-nm irradiation. Optical images (Fig. 6b–d) show that the emission can be seen as a blue-green color when samples are illuminated with an UV lamp with emission centered at 369 nm. The PL spectrum is broadened towards the lower energy side of the spectrum for longer ablation time due to the agglomeration of the CNDs. The excited carriers from the high-energy states in the smaller quantum to the low-energy states in the higher dots lead to an inhomogeneous broadening of the low energy tail. The increase in non-radiative recombination process due to the inhomogeneous broadening results in a decrease in the PL intensity with an increase in ablation time. The broadening results in the emergence of the green wavelength part of the spectrum. These results are corroborated through the optical pictures (Fig. 6) in which, for 150 s, a weak blue color is observed, being a kind of dark green for 900 s. Earlier studies on the origin of the PL in carbon dots demonstrated the correlation of the light emission with the size and homogeneity of the synthesized nanostructures [32]. A broad emission band was observed when a combination of CNDs and onion-shaped nanostructures were dispersed in water; however, the emission band was narrower for individual CNDs. In this work, it is observed that for 150 s of irradiation, a lower volume of CNDs, with similar size distribution is produced (Fig. 2d–f), which exhibit a narrower emission spectrum (Fig. 6a). The emission line width and the energy of emission at shorter irradiation time (Fig. 6a–c) are similar to that of the spectral characteristics of the individual CNDs in the previous report [32]. However, as the irradiation time is increased to 900 s, the inhomogeneous broadening due to agglomeration results in a broadening of the emission band (Fig. 5) which is similar to the CNDs were dispersed in water [32].

**Fig. 7:**
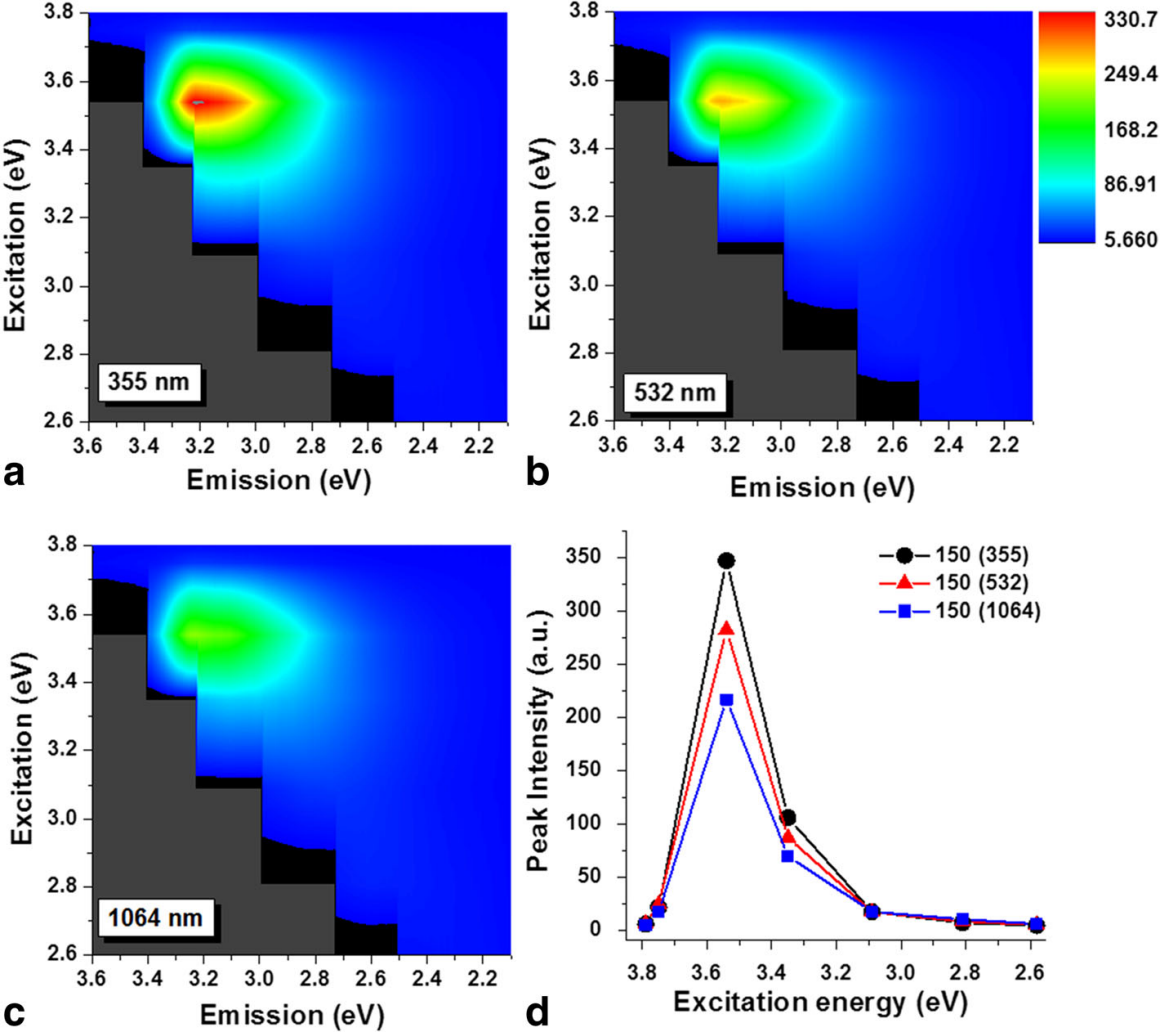
Excitation wavelength dependence (150 s of irradiation). Color plots of the CNDs solutions excited with different irradiation times for **a** 355, **b** 532, and **c** 1064 nm. **d** Maximum intensity recorded at different excitation wavelength

**Fig. 8:**
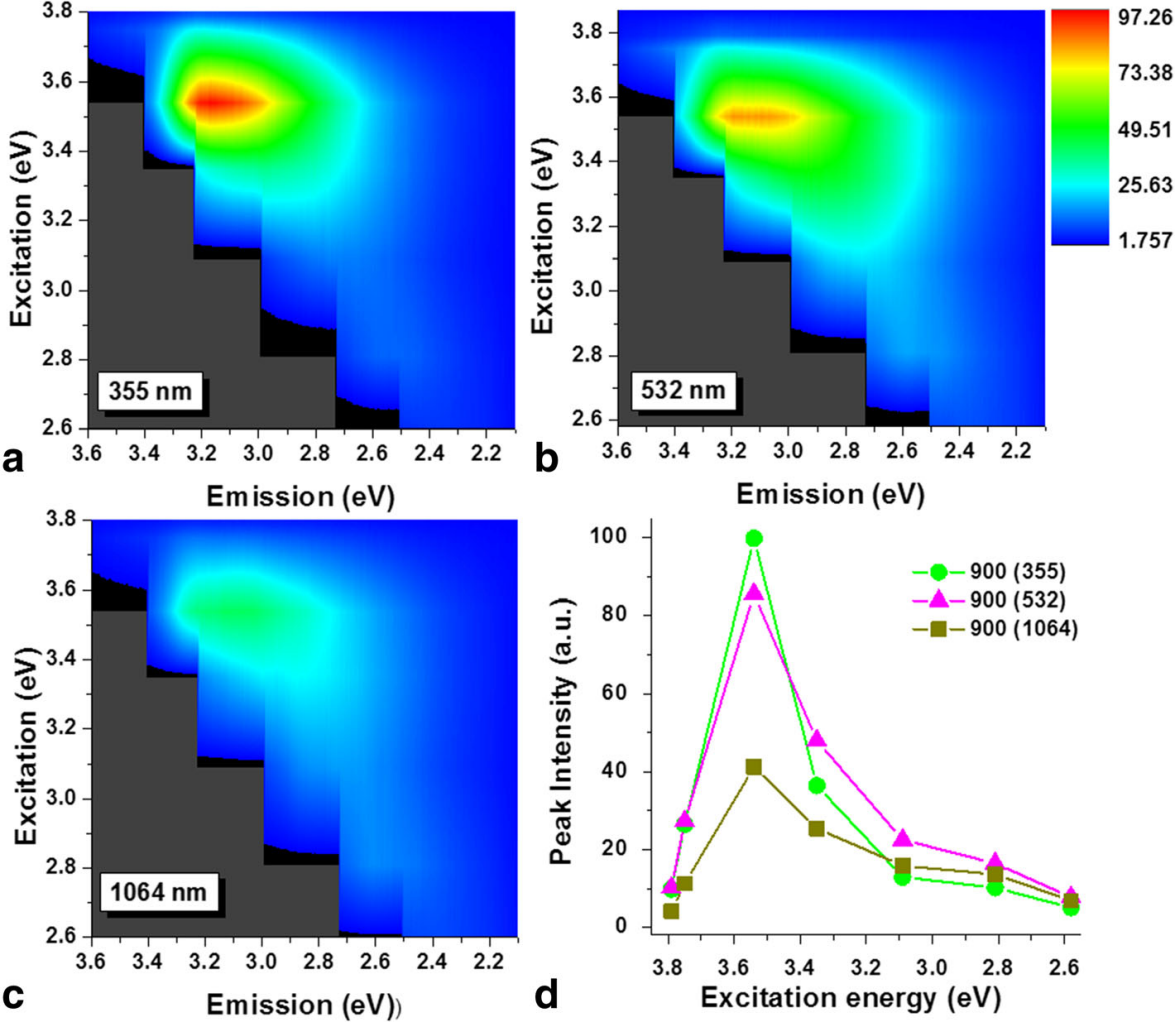
Excitation wavelength dependence (900 s of irradiation). Color plots of the CNDs solutions excited with different irradiation times for **a** 355, **b** 532, and **c** 1064 nm. **d** Maximum intensity recorded at different excitation wavelength

The effect of the excitation wavelength on the PL response of the CNDs solutions has also been studied. Figures 7 and 8, respectively, shows the emission map for ablation duration of 150 and 900 s, with excitation wavelengths ranging from 320 nm (3.87 eV) to 480 nm (2.58 eV) for the three wavelengths used for the synthesis. From color plots (a–c), it can be observed that the maximum PL emission was measured around 3.25 eV (380 nm) when the optical excitation energy was at 3.54 eV (350 nm). The maximum PL intensity with an excitation at 3.54 eV was observed to be sensitive to both, the irradiation time and the wavelength used for its synthesis. From Fig. 7a–c, it can be seen that the emission’s intensity at 3.25 eV is less for the CNDs solutions obtained with 1064 nm than for those with 355 and 532 nm when the irradiation time was 150 s. The same behavior was observed for the solutions synthesized with 900 s. However, in this case, as can be seen from Fig. 8a–c, the intensity decreases more than three times compared with that for 150 s (the intensity scale bar in the image was reduced from 0 to 330 to 0 to 100 for a better visualization). The variation in the PL intensity as a function of the excitation energy from samples obtained from the longer ablation time for each of the excitation wavelength is summarized in Fig. 7d and Fig. 8d. The emission characteristic dependent on excitation conditions has been reported in CNDs-like structures synthesized by ablating carbon solid targets [16, 18] or when fragmenting with laser pulses carbon powders suspended in liquid media [21–23].

The PL emission of CNDs is strongly influenced by the surface morphology and the crystalline defects which provide different defects or traps for the emitted photon. CNDs show spectrally a broad PL emission with a strong excitation wavelength dependency. It has been observed that the most common CNDs have a strong PL from blue to green color [5, 9], and our present work conforms to such results. However, the CNDs synthesized in our work possess optimal emission characteristics in the longer wavelength regime [5, 12].

**Fig. 9:**
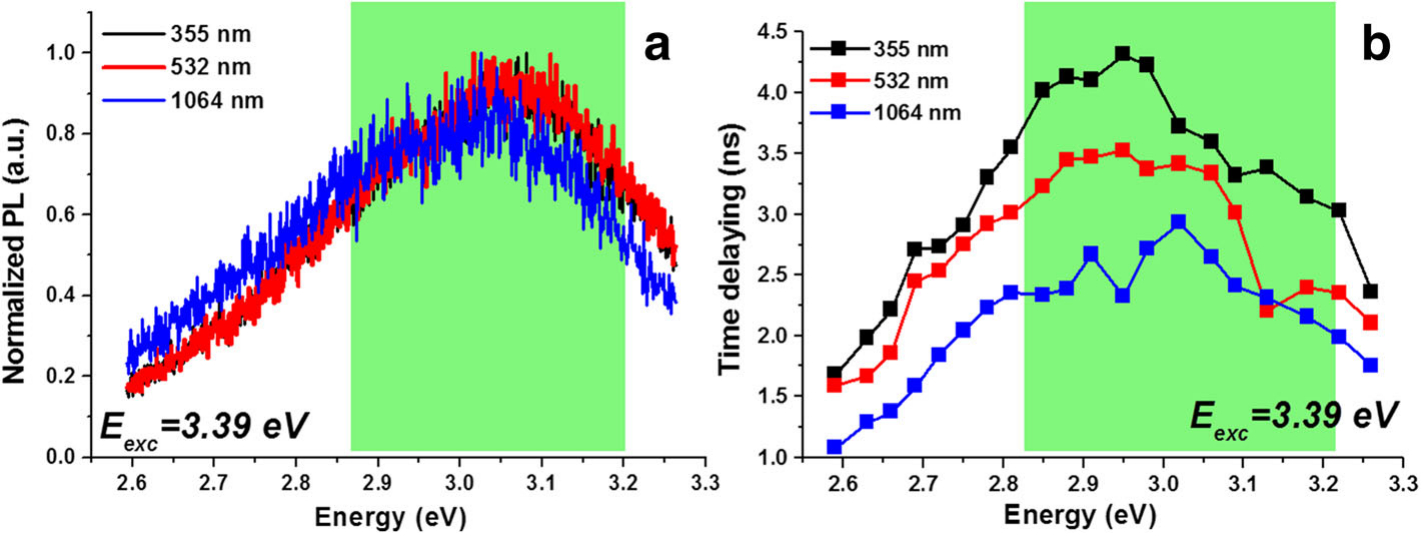
Time-resolved spectroscopy results exciting with 3.6 eV (345 nm). **a** Normalized PL spectrums for the three wavelengths of synthesis under 150 s of irradiation time. **b** PL lifetime measured as a function of emission energies for the samples in **a**

To estimate the contribution of the non-radiative recombination to the emission process, time-resolved spectroscopy was performed using a 3.6-eV excitation source. CNDs solutions synthesized with 150 s were analyzed. Figure 9a displays the normalized PL emission of the three solutions, which shows a broad band emission ranging from 2.6 to 3.3 eV, centered around 3.05 eV for 355 and 532 nm and slightly red-shifted to the low energy region for 1054 nm (green rectangle). This behavior is in good agreed with the previously PL discussed results.

A comparison of the photoluminescence lifetime at various energy states of the PL emission has been plotted in Fig. 9b. The PL lifetime has been measured for each of CNDs solution synthesized with 355, 532, and 1064 nm obtained with 150 s of irradiation time. The PL emission lifetime or the radiative recombination rate is longer for the 355 nm sample. The PL lifetime at the PL emission energy reduces with the ablation wavelength. The non-radiative recombination rate increases due to agglomeration and thus the PL lifetime is shorter for 1064-nm irradiation compared to 355-nm laser source. The integrated PL peak also red-shifts in samples with longer ablation time. This result is in perfectly agreement with the observed steady state PL response in Figs. 6 and 7. The agglomeration with the 1064-nm ablation results in non-radiative effects due to the increase in absorption by the dots in the solution. The longer PL lifetime at 355-nm ablation yield CNDs with higher light emission efficiencies.

## Conclusions

The laser ablation of carbon nanodots was performed using various laser sources and was optimized for the synthesis carried out over various ablation times. It is observed that the use of a short-wavelength UV laser source can yield carbon quantum dot-like structure whereas changing the laser ablation to the longer wavelength especially at 1064 nm results in agglomeration. The laser ablations sources with the skin depth confined to the surface of the target yield quantum dots with similar species. The highest spectral emission quality is observed for 150 s of irradiation as the PL intensity is most efficient with a minimum spectral line width. The emission intensity using the 355-nm wavelength ablation source has the brightest and most efficient emission compared to the infrared or visible light sources, particularly for the smallest irradiation time.
